# Panorama da COVID longa no Brasil: análise preliminar de um inquérito para pensar políticas de saúde

**DOI:** 10.1590/0102-311XPT094623

**Published:** 2024-04-29

**Authors:** Karina Barros Calife Batista, Michelle Vieira Fernandez, Lorena Guadalupe Barberia, Erica Tatiane da Silva, Vaneide Daciane Pedi, Bárbara Maia Lima Madeira Pontes, Gui Araujo, Rafael da Silveira Moreira, Marcos Pedrosa, Mariana Pastorello Verotti, Claudio Maierovitch Pessanha Henriques, Anna Catharina Florêncio, Melania Maria Ramos de Amorim

**Affiliations:** 1 Faculdade de Medicina, Universidade de São Paulo, São Paulo, Brasil.; 2 Faculdade de Ciências Médicas da Santa Casa de São Paulo, São Paulo, Brasil.; 3 Universidade de Brasília, Brasília, Brasil.; 4 Instituto Aggeu Magalhães, Fundação Oswaldo Cruz, Recife, Brasil.; 5 Faculdade de Filosofia, Letras e Ciências Humanas, Universidade de São Paulo, São Paulo, Brasil.; 6 Fiocruz Brasília, Fundação Oswaldo Cruz, Brasília, Brasil.; 7 Ministério da Saúde, Brasília, Brasil.; 8 Swansea University, Swansea, U. K.; 9 Faculdade de Medicina, Universidade Federal de Pernambuco, Recife, Brasil.; 10 Instituto de Medicina Integral Professor Fernando Figueira, Recife, Brasil.

**Keywords:** Síndrome Pós-COVID-19 Aguda, COVID-19, Política Pública, Sintomas, Post-Acute COVID-19 Syndrome, COVID-19, Public Policy, Signs and Symptoms, Síndrome Post Agudo de COVID-19, COVID-19, Política Pública, Síntomas

## Abstract

Caracterizada por sintomas que permanecem ou aparecem pela primeira vez em até três meses após a infecção pelo SARS-CoV-2, a COVID longa pode se manifestar de diferentes formas, inclusive entre casos não hospitalizados ou assintomáticos. Nesse sentido, este artigo apresenta um panorama da COVID longa no Brasil, com ênfase no diagnóstico, nos sintomas e nos desafios para a nova gestão da saúde. Foram utilizados dados de um estudo realizado com objetivo de investigar a COVID longa em pessoas acometidas pela COVID-19, com dados originais de um inquérito com indivíduos brasileiros adultos (18 anos ou mais) que tiveram COVID-19, coletados entre 14 de março e 14 de abril de 2022, por meio de questionário divulgado em redes sociais. O questionário abordou características sociodemográficas, histórico de infecções por COVID-19, vacinação contra a doença, investigação da situação de saúde e da qualidade de vida antes e após a COVID-19, além da busca e acesso a tratamento. Dos 1.728 respondentes, 720 foram considerados elegíveis para a análise. Desses, 496 (69%) tiveram COVID longa. Os indivíduos com COVID longa reportaram manifestações clínicas como ansiedade (80%), perda de memória (78%), dor generalizada (77%), falta de atenção (75%), fadiga (73%), queda de cabelo (71%), alterações de sono (70%), alterações de humor (62%), indisposição (60%) e dor nas articulações (59%). A maioria procurou os serviços de saúde durante e após a fase aguda de COVID-19 (94% e 80%, respectivamente), o que representa a necessidade de estruturar o sistema de saúde para atender esses pacientes.

## Abstract (en)

Characterized by symptoms that remain or appear for the first time within three months of SARS-CoV-2 infection, long COVID can manifest itself in different ways, including in non-hospitalized or asymptomatic cases. Thus, this study offers an overview of long COVID in Brazil, especially of its diagnosis, symptoms, and challenges for new health management. Data from a study that investigated long COVID in people affected by COVID-19 were used. These original data stem from a survey with adult Brazilians (aged 18 years or older) who had COVID-19 that collected information from March 14 to April 14, 2022, by a questionnaire on social media. The questionnaire addressed sociodemographic characteristics, history of COVID-19 infections, vaccination against the disease, investigation of health status and quality of life before and after COVID-19, and search and access to treatment. Of the 1,728 respondents, 720 were considered eligible for analysis, of which 496 (69%) had long COVID. Individuals with long COVID reported clinical manifestations such as anxiety (80%), memory loss (78%), generalized pain (77%), lack of attention (75%), fatigue (73%), hair loss (71%), sleep changes (70%), mood swings (62%), malaise (60%), and joint pain (59%). Most sought health services during and after the acute phase of COVID-19 (94 and 80%, respectively), representing the need to structure the healthcare system for these patients.

## Abstract (es)

Caracterizado por síntomas que permanecen o aparecen por primera vez dentro de los tres meses posteriores a la infección por SARS-CoV-2, la COVID larga puede manifestarse de diferentes formas, incluso entre casos no hospitalizados o asintomáticos. En este sentido, este artículo presenta un panorama la COVID larga en Brasil, con énfasis en el diagnóstico, los síntomas y los desafíos para la nueva gestión de la salud. Se utilizaron datos de una encuesta realizada para investigar la COVID larga en personas afectadas por COVID-19. Se trata de datos originales de una encuesta con individuos brasileños adultos (18 años o más), que tuvieron COVID-19, con datos recolectados entre el 14 de marzo y el 14 de abril de 2022, por medio de un cuestionario divulgado en las redes sociales. El cuestionario abordó características sociodemográficas, historial de infecciones por COVID-19, vacunación contra la enfermedad, investigación de la situación de salud y de la calidad de vida antes y después de COVID-19, además de la búsqueda y acceso a tratamiento. De los 1.728 encuestados, 720 fueron considerados elegibles para el análisis. De ellos, 496 (69%) tenían COVID larga. Las personas con COVID larga informaron manifestaciones clínicas como ansiedad (80%), pérdida de memoria (78%), dolor generalizado (77%), falta de atención (75%), fatiga (73%), pérdida de cabello (71%), cambios en el sueño (70%), cambios de humor (62%), malestar (60%) y dolor en las articulaciones (59%). La mayoría recurrió a los servicios de salud durante y después de la fase aguda de COVID-19 (94% y 80%, respectivamente), lo que representa la necesidad de estructurar el sistema de salud para atender a estos pacientes.

Quatro anos após o início da pandemia de COVID-19, sabemos que o impacto da infecção pelo SARS-CoV-2 vai muito além da fase aguda da doença. De 10 a 20% dos que se recuperam da COVID-19 apresentam sintomas que impactam na saúde e na qualidade de vida, em decorrência da COVID longa ou condições pós-COVID-19 ^1^. A COVID longa caracteriza-se por sintomas que permanecem ou aparecem pela primeira vez em até três meses após a infecção pela COVID-19, que duram por pelo menos dois meses e que não podem ser explicados por outros motivos, conforme definição adotada pela Organização Mundial da Saúde (OMS) ^1^. Manifesta-se de diferentes formas, inclusive entre casos não hospitalizados ou assintomáticos ^2^.

Mais de 200 sintomas foram associados à COVID longa, não se restringindo a manifestações respiratórias ^3^. Seu tratamento depende dos órgãos e sistemas envolvidos, aumentando a demanda por especialistas e equipes multidisciplinares (clínicos, neurologistas, cardiologistas, pneumologistas, psiquiatras, psicólogos, fisioterapeutas, entre outros) ^4^, de forma semelhante às doenças crônicas, nas redes de atenção à saúde e nos complexos reguladores ^5^^,^^6^^,^^7^.

Até outubro de 2023, com mais de 600 milhões de casos de COVID-19 confirmados no mundo, incluindo mais de 6,8 milhões de óbitos, o Brasil foi responsável por aproximadamente 37 milhões desses casos e mais de 699 mil dos óbitos registrados, configurando um dos piores cenários da pandemia na América Latina ^8^. Esse contexto alerta para um importante problema de saúde pública a ser enfrentado: casos com sintomas duradouros ou recorrentes da COVID-19, mesmo após a fase aguda da doença, que necessitam de cuidados especializados para a recuperação da saúde e da qualidade de vida ^5^^,^^6^^,^^7^.

Apresentamos, neste artigo, um panorama da COVID longa no Brasil, no que se refere a diagnóstico, sintomas e desafios para a nova gestão da saúde. Foram utilizados dados de um estudo realizado com o objetivo de investigar o impacto da COVID longa em pessoas acometidas pela COVID-19. São dados originais de um inquérito com indivíduos brasileiros adultos (18 anos ou mais) que tiveram COVID-19, coletados entre 14 de março e 14 de abril de 2022, por meio de questionário divulgado em redes sociais. O questionário *online* compreendeu características sociodemográficas, histórico de infecções por COVID-19, vacinação contra a doença, investigação da situação de saúde e da qualidade de vida antes e após a COVID-19, além da busca e acesso a serviços de saúde.

O estudo apresenta algumas limitações que devem ser consideradas na interpretação dos resultados. A primeira delas é o método de amostragem utilizado. O inquérito foi divulgado em redes sociais, o que pode ter limitado a sua representatividade da população brasileira. A exclusão de pessoas que não usam redes sociais ou não têm acesso à internet representa uma lacuna potencial, impedindo a incorporação de perspectivas de grupos que podem enfrentar desafios distintos em relação à COVID-19. Além disso, esse tipo de coleta levanta a possibilidade de viés de autosseleção, já que indivíduos com experiências mais notáveis ou persistentes da COVID-19 podem ter sido mais propensos a participar. O fato de a pesquisa ter se concentrado em adultos com diagnóstico confirmado por reação de transcriptase reversa seguida de reação em cadeia da polimerase (RT-PCR) pode excluir aqueles que tiveram a doença com manifestações mais leves ou que não buscaram testagem. Por fim, outra limitação da pesquisa diz respeito ao tamanho da amostra, que, embora composta por 1.728 participantes, não captura a diversidade da população brasileira. Essas considerações ressaltam a importância de interpretar os resultados com cautela, reconhecendo as limitações inerentes ao método de amostragem e ao tamanho da amostra, e sublinham a necessidade de abordagens mais abrangentes em futuras pesquisas sobre as condições pós-COVID-19 no Brasil.

Dos 1.728 participantes, 1.230 tiveram o diagnóstico de COVID-19 confirmado por RT-PCR. Desses, 720 tiveram o quadro agudo da doença há pelo menos três meses do momento da sua participação na pesquisa. Entre esses 720, 496 (69%) disseram não ter se recuperado da COVID-19 e foram, portanto, considerados como casos de COVID longa para fins de análise neste estudo. A proporção da ocorrência de COVID longa entre os indivíduos do sexo feminino (88%) foi maior do que entre os indivíduos do sexo masculino (52%), com χ^2^ = 17,84 (n = 720, p < 0,001). Do total de indivíduos com COVID longa, 88% eram do sexo feminino e 39% tinham entre 25 e 40 anos de idade.

Dos 720 indivíduos com COVID-19 diagnosticados por RT-PCR que tiveram o quadro agudo da doença, 27% foram vacinados contra a COVID-19. A partir desse dado, a ocorrência de COVID longa foi maior entre os não vacinados (72,5%) em comparação com os que receberam vacinação contra COVID-19 (59%), com χ^2^ = 11,87 (n = 720, p < 0,001). Foram elencadas mais de 50 manifestações clínicas associadas à COVID longa. Essas foram classificadas em: cardiovasculares ou de coagulação, dermatológicas, endócrino-metabólicas, gastrointestinais, músculo-esqueléticas, neurológicas, de saúde mental, renais e respiratórias, além de sintomas gerais (Figura 1). Destaca-se a diversidade de órgãos e sistemas do corpo envolvidos nos relatos dos participantes com COVID longa, sendo que os principais sintomas foram ansiedade (80%), perda de memória (78%), dor generalizada (77%), falta de atenção (75%), fadiga (73%), queda de cabelo (71%), alterações de sono (70%), alterações de humor (62%), indisposição (60%) e dor nas articulações (59%).

**Figura 1:**
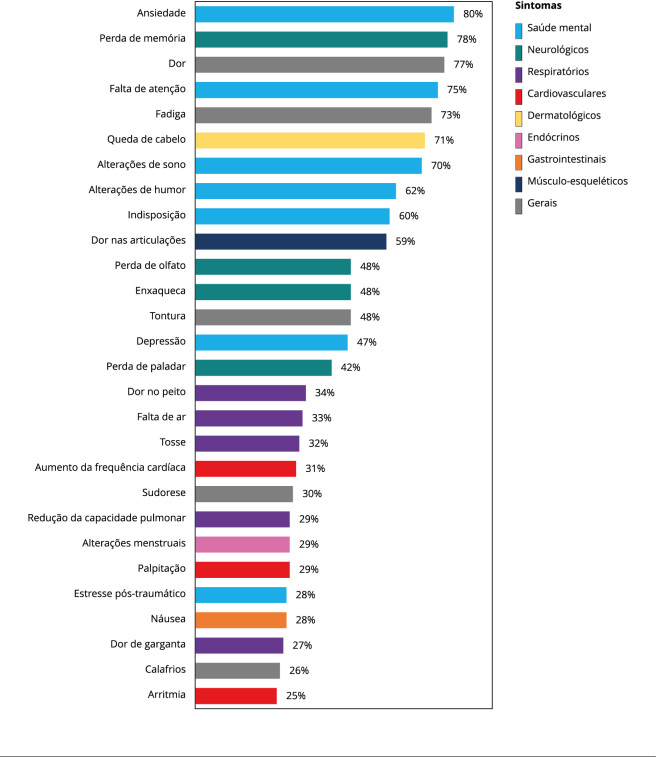
Principais sintomas relatados pelos participantes do inquérito com COVID longa.

Fonte: elaboração própria.

A busca pelos serviços de saúde durante a fase aguda da COVID-19 foi reportada por 94% dos indivíduos com COVID longa. Além disso, 23% precisaram de internação hospitalar. Por outro lado, apenas 6% dos respondentes que tiveram COVID longa tiveram doença assintomática ou leve. Após a fase aguda da COVID-19, 80% dos respondentes com COVID longa procuraram serviços de saúde, sendo unidades básicas de saúde ou serviços da rede particular, por meio de consultas presenciais ou teleatendimentos.

No Brasil, ao longo do governo Jair Bolsonaro (2019-2022), chamou a atenção a pouca atuação do Governo Federal no monitoramento da COVID longa e a ausência de estratégias de cuidado e campanhas para alertar a população sobre a COVID-19, de forma geral, e suas consequências. A partir de 2022, recursos específicos foram destinados para as ações e os serviços voltados para pessoas com sintomas pós-COVID na atenção primária à saúde (APS), por meio da *Portaria nº 377/2022*^9^. Os recursos foram distribuídos seguindo critérios estabelecidos na própria Portaria, classificando os municípios em perfis “alto”, “médio”, e “baixo”. No entanto, apesar da destinação de recursos, não se observa uma estruturação do sistema de saúde para o cuidado de paciente com COVID longa.

Até 2022, a demora para o estabelecimento de diretrizes por parte do Governo Federal levou à ação descoordenada de estados e municípios, sem centralizar um plano de ação e cuidado para o território nacional, criando desigualdades na atenção à saúde. Apesar da responsabilidade solidária entre os entes federativos para garantir adequada atenção à saúde, a atuação do Ministério da Saúde no estabelecimento de diretrizes e protocolos é fundamental à atuação dos outros entes federados. Por isso, até 2022, observamos algumas experiências pontuais desenvolvidas por estados ou municípios ^10^^,^^11^^,^^12^, compreendendo linhas de ação, para mitigar os sintomas prolongados da COVID-19. Em dezembro de 2022, o Grupo Técnico de Saúde da Comissão de Transição Governamental relata insuficiência e imprecisão de dados sobre a temática no governo anterior ^13^. Aponta-se, ainda, a necessidade da criação de políticas públicas específicas para o tratamento de sintomas pós-COVID-19.

Diante da lacuna deixada pela inação do Governo Federal durante o governo Bolsonaro, o Parlamento, por meio da atuação de deputados federais, lançou algumas iniciativas que tocam na situação de pacientes no pós-COVID-19. O *Projeto de Lei nº 5.026/2020*, do deputado Célio Silveira (Partido da Social Democracia Brasileira - PSDB/GO), debate a assistência aos pacientes que tiveram COVID-19, mesmo após a alta hospitalar. Outras iniciativas foram criadas, nesse âmbito, e apensadas a esse primeiro projeto: o *Projeto de Lei nº 1.487/2021*, do deputado Altineu Côrtes (Partido Liberal - PL/RJ), que incluiria as pessoas com sequelas com sintomas prolongados da COVID-19 ao Benefício de Prestação Continuada (BPC); o *Projeto de Lei nº 2.369/2021* do deputado Nivaldo Albuquerque (Republicanos/AL), que trata da criação de um programa específico para a síndrome da COVID longa e estímulo a pesquisas sobre a temática; e o *Projeto de Lei nº 901/2021*, do deputado Zeca Dirceu (Partido dos Trabalhadores - PT/PR), que assegura, no âmbito do Sistema Único de Saúde (SUS), a reabilitação de pessoas com sequelas decorrentes da COVID-19. O *Projeto de Lei nº 5.026/2020*, que anexou todos os outros, está atualmente na Comissão de Finanças e Tributação (CFT) da Câmara dos Deputados, após ter sido aprovado com alterações na Comissão de Saúde. Em seguida, deverá ser apreciada nas Comissões de Seguridade Social e Família, Cidadania e Constituição e Justiça.

Os achados desta pesquisa evidenciam a incidência da COVID longa na vida das pessoas acometidas pela COVID-19. Nesse sentido, é necessário que o Ministério da Saúde estabeleça mecanismos para se aproximar da população com COVID longa e construa protocolos de monitoramento dos casos e avaliação de consequências na vida das pessoas. Dessa forma, o Governo Federal geraria subsídios para a execução de condutas eficazes e informadas por evidências para a atuação de profissionais da saúde e gestores públicos. Fica evidente a importância da retomada da coordenação das políticas de saúde pelo Ministério da Saúde e da atuação de estados e municípios na gestão de serviços, no planejamento em saúde e na organização das redes de atenção para lidar com os impactos produzidos por essa e por outras emergências em saúde pública que venham a surgir. Além disso, dada a complexidade do cuidado necessário a pacientes com COVID longa, destacamos a necessidade do cuidado integral desses pacientes. Nesse sentido, a APS pode atuar de forma central para uma melhor abordagem inicial, no acolhimento e seguimento de pessoas com COVID longa.

É fundamental esclarecer a importância da mobilização de diferentes áreas de políticas públicas, com uma articulação entre os diferentes entes subnacionais em temas de desenvolvimento social, previdência e trabalho. Por fim, é imperativo levar em consideração o financiamento para esses cuidados no contexto de subfinanciamento do SUS e da saúde no Brasil ^14^. Apesar da emergência sanitária ter permitido uma flexibilização das regras fiscais e de alocação de recursos em forma de créditos adicionais extraordinários para o Ministério da Saúde, a pandemia da COVID-19 evidencia os atuais problemas ligados ao financiamento da saúde no Brasil, em especial a partir da aprovação da *Emenda Constitucional nº 95/2016*^15^.
